# Stimulus‐induced rotary saturation imaging of visually evoked response: A pilot study

**DOI:** 10.1002/nbm.5280

**Published:** 2024-11-04

**Authors:** Milena Capiglioni, Roland Beisteiner, Pedro Lima Cardoso, Federico Turco, Baudouin Jin, Claus Kiefer, Simon Daniel Robinson, Andrea Federspiel, Siegfried Trattnig, Roland Wiest

**Affiliations:** ^1^ Support Centre for Advanced Neuroimaging Institute for Diagnostic and Interventional Neuroradiology, University of Bern Bern Switzerland; ^2^ Graduate School for Cellular and Biomedical Sciences (GCB) University of Bern Bern Switzerland; ^3^ Department of Neurology, Laboratory for Functional Brain Diagnostics and Therapy, High Field MR Center Medical University of Vienna Wien Austria; ^4^ High Field MR Center, Department of Biomedical Imaging and Image‐guided Therapy Medical University of Vienna Vienna Austria

**Keywords:** functional magnetic resonance imaging, neuroelectric oscillations, neuronal activity, non‐BOLD fMRI, spin‐lock, visual stimulation

## Abstract

Spin‐lock (SL) pulses have been proposed to directly detect neuronal activity otherwise inaccessible through standard functional magnetic resonance imaging. However, the practical limits of this technique remain unexplored. Key challenges in SL‐based detection include ultra‐weak signal variations, sensitivity to magnetic field inhomogeneities, and potential contamination from blood oxygen level‐dependent effects, all of which hinder the reliable isolation of neuronal signals. This pilot study evaluates the performance of the stimulus‐induced rotary saturation (SIRS) technique to map visual stimulation response in the human cortex. A rotary echo spin‐lock (RESL) preparation followed by a 2D echo planar imaging readout was used to investigate 12 healthy subjects at rest and during continuous exposure to 8 Hz flickering light. The SL amplitude was fixed to the target neuroelectric oscillations at that frequency. The signal variance was used as contrast metric, and two alternative post‐processing pipelines (regression‐filtering‐rectification and normalized subtraction) were statistically evaluated. Higher variance in the SL signal was detected in four of the 12 subjects. Although group‐level analysis indicated activation in the occipital pole, analysis of variance revealed that this difference was not statistically significant, highlighting the need for comparable control measures and more robust preparations. Further optimization in sensitivity and robustness is required to noninvasively detect physiological neuroelectric activity in the human brain.

## Graphical abstract

This study investigates the stimulus‐induced rotary saturation (SIRS) signal response in healthy volunteers under visual stimulation and proposes an experimental and post‐processing pipeline to distinguish rotary saturation contrast from physiological and hemodynamic confounds. SIRS detected activation in the visual cortex at the group level, but the proposed acquisition could not reliably detect activation at the individual subject level. Further improvements in sequence and experimental protocol are suggested.

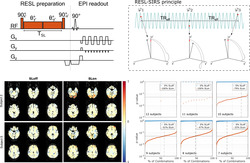

**Abbreviations**
NS: normalized subtraction
RESL: rotary echo spin‐lock
RFR: regression‐filtering‐rectification
SIRS: stimulus‐induced rotary saturation

## 1 INTRODUCTION

The neuroimaging community has shown a notable emphasis on advancing noninvasive methods to directly detect neuronal activity. The focus aims to complement existing techniques that either measure neuronal activation indirectly through blood oxygen level‐dependent (BOLD) contrast^1^, ^2^ or have insufficient spatial specificity such as electroencephalography (EEG)^3^ or magnetoencephalography (MEG).^4^ Within the magnetic resonance (MR) community, some remarkable examples include functional magnetic resonance spectroscopy,^5^ functional diffusion‐weighted imaging,^6^, ^7^ and the novel (and currently controversial) direct imaging of neuronal activity (DIANA) method.^8^, ^9^, ^10^, ^11^ Another non‐BOLD MR approach aimed to detect the phase changes generated by the fluctuation of the B0 field induced by the neuronal currents.^12^, ^13^ Closer to the latter, this pilot study centers in a promising method that targets ultraweak transient magnetic fields induced by axonal currents through a spin‐lock (SL) prepared MR sequence named stimulus‐induced rotary saturation (SIRS).^14^, ^15^

The SIRS contrast is based on the resonance effect that occurs when the frequency of a neurally induced oscillatory field matches the SL‐induced frequency. Therefore, electromagnetic activity can be directly detected while maintaining spatial resolution in the range of a BOLD acquisition. Since its introduction in 2008,^14^ simulations, phantom, and human studies have been performed to increase the method's sensitivity and robustness.^15^, ^16^, ^17^, ^18^, ^19^, ^20^

A notable advantage of the SIRS technique is its frequency selectivity, which can potentially target pathologies displaying a specific frequency of brain activity.^21^, ^22^, ^23^, ^24^ Previous SIRS studies using a rotary echo spin‐lock preparation (RESL‐SIRS) reported to have successfully localized the *seizure onset zone* in pre‐surgical epilepsy patients,^25^ detected higher contrast in first seizure patients,^26^ and epileptogenic tissue in dogs with idiopathic epilepsy.^27^ Because epilepsy activity is associated with exceptionally increased and hypersynchronous electrical activity,^28^, ^29^, ^30^ it remains unknown whether RESL‐SIRS can effectively map neuroelectric oscillations induced by physiological activity in the healthy brain.^31^ Consequently, this study aimed to assess the robustness and sensitivity of the RESL‐SIRS sequence in the context of healthy individuals subjected to visual stimulation.

In this article, we analyzed RESL‐SIRS data from healthy volunteers at rest and during continuous visual stimulation at the target SL frequency. We used two previously proposed processing pipelines to clean the data from low‐frequency physiological noise and hemodynamic confounds. We conducted a statistical assessment to determine if there are discernible differences in image contrast metrics between the two stimulation conditions. Additionally, this pilot study identifies weaknesses and strengths of the protocol and processing pipelines used, aiming to provide guidance in methodological aspects and support design adoptions for future clinical studies.

## 2 METHODS

### 2.1 RESL‐SIRS principle and protocol design

**FIGURE 1:**
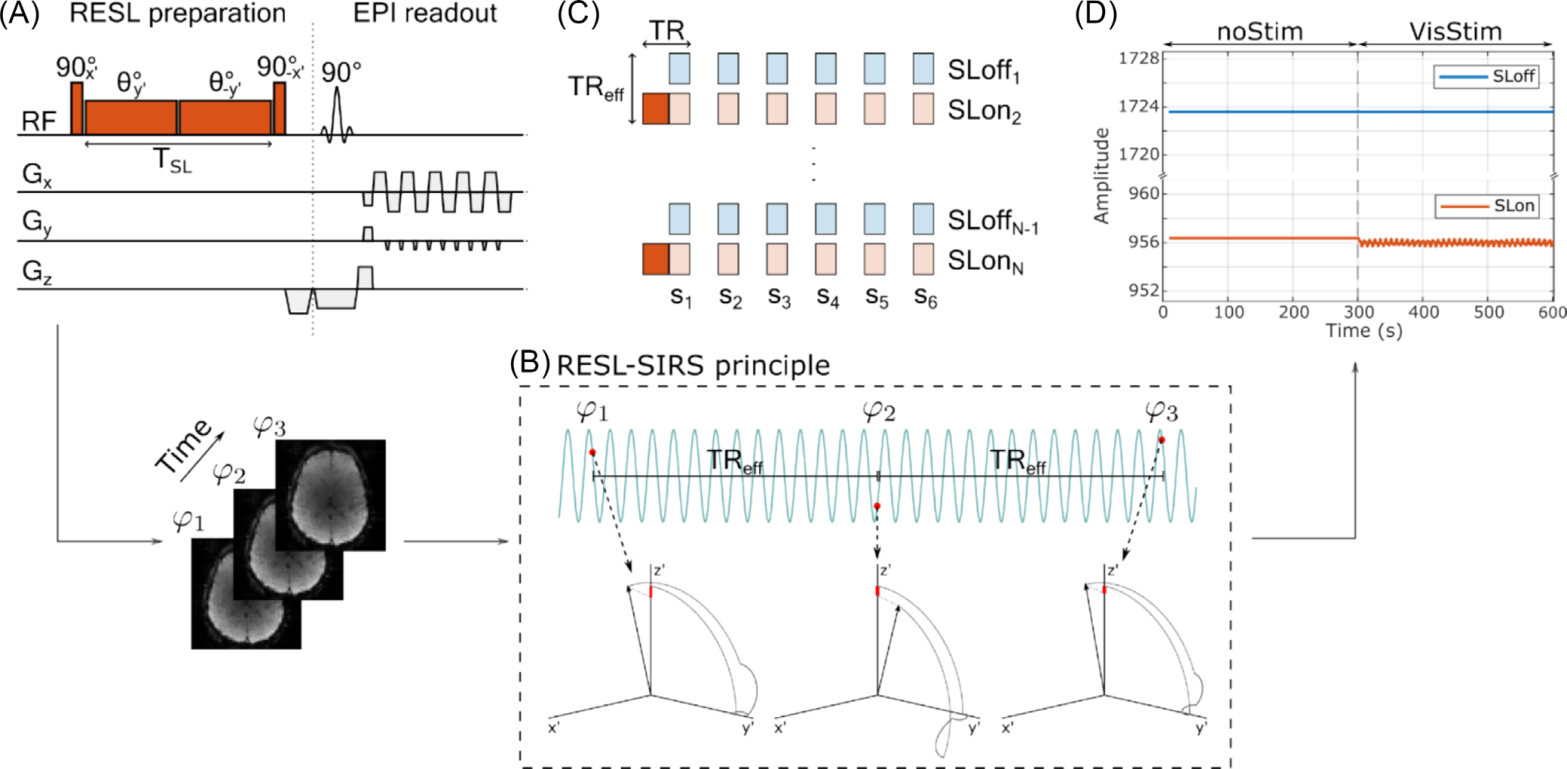
Neuronal current imaging sequence. (A) Nonselective rotary‐echo spin‐lock preparation (RESL) is followed by consecutive slice‐selective 90° excitations and 2D echo‐planar readouts. (B) The repetition of SL‐prepared acquisitions samples various initial phases of the target field, leading to different magnetization dynamics during preparation and consequently different saturation amplitudes. (C) Acquisition protocol for six slices alternating between nonprepared and RESL‐prepared volume readouts. The effective repetition time is the period between two prepared or nonprepared acquisitions. (D) Bloch simulation of RESL‐SIRS full acquisition where only the rotary saturation effect is considered. RESL, rotary echo spin‐lock; SIRS, stimulus‐induced rotary saturation; SL, spin‐lock.

The nonselective RESL preparation used in this study contains four radiofrequency (RF) pulses. The first 90° pulse tilts the magnetization to the transversal plane, where it is locked by two successive RF pulses of opposite phase with total added duration $T_{\mathrm{SL}}$. The amplitude of the SL pulses ($B_{\mathrm{SL}}$) defines the oscillation frequency of the magnetization around the SL axis as $F_{\mathrm{SL}}=2\pi \gamma B_{\mathrm{SL}}$. Each SL half induces a rotation angle $\theta =\pi F_{\mathrm{SL}}T_{\mathrm{SL}}$ (Figure 1A). If during the SL pulse, an external magnetic field along *z* oscillates with $F_{\mathrm{SL}}$, it will act as an excitation pulse, deviating the magnetization from the SL direction.^14^ The last 90° pulse tilts the magnetization back to the longitudinal axis. The opposite phase of the two SL pulses makes this preparation robust against transmit field ($B_{1}$) imperfections, while remaining susceptible to inhomogeneities of the static field $B_{0}.$ ^18^ Figure 1B displays the magnetization dynamic for different initial phases of the target field when ignoring relaxation. The final longitudinal amplitude decreases due to the rotary saturation contrast. After preparation, a spoiler gradient eliminates the remaining transverse magnetization. Volume acquisition is performed with six successive 2D echo‐planar images (EPI) covering the visual cortex.

One fundamental challenge within the SIRS technique is the question of whether it genuinely detects neural oscillations without being affected by hemodynamic contaminations. To contemplate the presence of such effect, successive measurements alternate between RESL prepared (SLon) and nonprepared (SLoff) acquisitions, as was previously suggested.^25^ The rationale is that hemodynamic effects and low‐frequency confounds will be captured by the common EPI readout and can be filtered in the processing stage. The effective repetition time TR_eff_ is the period between two prepared or nonprepared acquisitions (Figure 1C). Therefore, each repetition samples different initial phases ($\varphi_{N})$ of the target oscillating field (defined by the relationship between $F_{\mathrm{SL}}$ and TR_eff_), and the amount of saturation changes for each SL repetition^18^ (Figure 1B). More details on the timing and acquisition parameters are specified in Supplementary material 1.

### 2.2 Data acquisition

The study was approved by the Ethics Committee of the Medical University of Vienna. Twelve healthy volunteers (age 18–65 years) participated in the study and provided written informed consent. Exclusion criteria included significant illness, pregnancy, morbid obesity, claustrophobia, and pacemakers. Participants were trained to keep their eyes open during the whole experiment.

All MRI scans were performed on a 3T PRISMA scanner (Siemens, Germany) with a 64‐channel head coil. High‐resolution images were acquired using a standard MP2RAGE sequence with TR/TE = 5000/2.98 ms; inversion times TI1/TI2 = 700/2500 ms; 160 slices; voxel‐size 0.5 × 0.5 × 1 mm. For the SIRS scans, imaging parameters were TR = 139.55 ms, TR_eff_ = 1674.6 ms, TE = 29 ms, six slices, voxel‐size = 3.6 × 3.6 × 4 mm, and matrix size = 64 × 64. In all SLon acquisitions, $F_{\mathrm{SL}}$ was chosen at 8 Hz, a frequency considered optimal for visual stimulation paradigms in MEG and functional magnetic resonance imaging research.^32^, ^33^ The $T_{\mathrm{SL}}$ was set to 70 ms to align with prior research demonstrating positive detection with the RESL‐SIRS sequence.^25^, ^27^ Slice selection was standardized for all subjects placed in supine position by aligning sagittal slices with the calcarine sulcus in the coronal plane. Six slices were acquired sequentially with a 100% gap between them to encompass most of the occipital pole.

Each volunteer underwent two RESL‐SIRS acquisitions (5 min each), including 180 SLon and 180 SLoff measurements. In the first acquisition, the volunteer remained at rest with the lights off (noStim condition). During the second acquisition, a stroboscope (Dawe stroboscope type 1214B, Grossegger & Drbal) was placed behind the subject and projected onto MRI Prism Glasses. The stimulation frequency was set to 8 Hz and applied continuously throughout the second acquisition (VisStim condition). Two time courses are recorded for each voxel, SLoff (no Stim and VisStim) and SLon (noStim and VisStim).

Figure 1D shows a Bloch simulation with the same acquisition parameters used in this study for a voxel in the first slice where a 4‐nT resonant field is present. The simulator was implemented following the procedure outlined in reference.^18^ While the simulator does not consider hemodynamic effects, such effect would manifest as a change in the signal mean of both SLon and SLoff acquisition during stimulation. Instead, the rotary saturation manifests as an increased variance during the stimulation period. This distinction is critical for understanding the implementation of the processing pipelines outlined in the next section.

### 2.3 Data analysis

Image preprocessing prior to signal analysis included motion correction using MCFLIRT (FSL version 6.04)^34^ and within‐subject coregistration with the high‐resolution image using SPM 12.^35^ The hypothesis of this work is that SL‐prepared acquisitions exhibit higher variance during visual stimulation^18^, ^36^ and that other signal confounds (i.e., BOLD effect) are encoded in both SLon and SLoff acquisitions by the common EPI readout. Building upon previous experimental findings, two alternative post‐processing pipelines based on the normalized subtraction (NS)^25^ and the regression‐filtering‐rectification (RFR)^36^ procedures were implemented to compare signal variance in resting and stimulated conditions.

#### 2.3.1 RFR analysis

We adapted the RFR procedure proposed by Truong et al.,^36^ which originally involved comparing power at the task‐based frequency of a block design, to our experimental protocol defined by only one rest and one stimulation period. First, high‐resolution anatomical images were segmented into the 74 cortical parcels of the Destrieux atlas^37^ using DL + DiReCT.^38^ After coregistration, the mean of each parcel in functional space was subtracted from each voxel time course within it (regression). Second, a high‐pass filter with a 0.1‐Hz cutoff (below the TR) removed the remaining slow physiological confounds (filtering). Finally, the mean of each voxel in time was subtracted from the voxel time course, and the absolute value calculated (rectification). The mean of the rectified signal is proportional to the variance of the RFR output and serves as functional activation metric. The result is an activation map for each of the four signals acquired for each subject.

Individual activation was evaluated by analyzing the value distribution of all voxels within the anatomically defined occipital pole (V1 ROI). Additionally, following the analysis presented by Truong et al., we performed statistical group analysis across 12, 11, 10, 9, 8, and 7 subjects by averaging the contrast within the voxels in the V1 ROI.^36^ For both individual and group analysis, a one‐tailed paired *t*‐test (*p* < 0.05) was conducted to compare the visual stimulation vs. rest series for both SLon and SLoff acquisitions.

The previous individual and group analysis allows comparison with the other only study performed with visual stimulation and the SL principle.^33^ Additionally, we performed a two‐way analysis of variance (ANOVA) to assess the relative importance and interaction of two factors on the metric of interest. The first factor was the presence of SL preparation with levels “Slon” and “SLoff.” The second factor was visual stimulation, with levels “noStim” and “VisStim.” Due to the small field of view (FOV) in the acquisition, normalizing to standard space led to missing data for some voxels across subjects. To address this, we performed the ANOVA analysis only within Destrieux atlas parcels that had data available from all 12 subjects. This parcel‐wise analysis produced *F* and *p* values for each factor (SL and visual stimulation) and their interaction within each atlas parcel.

#### 2.3.2 NS analysis

We adapted the NS method originally proposed for alternated SLon and SLoff acquisitions.^25^ This approach assumes that low‐frequency noise is equally encoded in two consecutive measurements. Therefore, $\mathrm{NS}\left(t\right)$, computed as $\text{SLon}\left(t\right)–\text{SLoff}\left(t\right)\frac{\text{SLon}\left(0\right)}{\text{SLoff}\;\left(0\right)}$, can in principle remove the noise encoded in the common EPI readout. $\text{SLon}\left(0\right)$ and $\text{SLoff}\left(0\right)$ represent the first time point after the dummy acquisitions (see Supplementary Material 2 for more details). In the original NS work, the contrast is calculated as $\max\left(z_{s}\right)-z_{s}$, where $z_{s}$ is the *z*‐score module of the NS, used to highlight short‐sporadic rotary effects as expected in epileptic activity. In this work, we adapted the contrast calculation to work with our continuous rest and stimulation periods by generating contrast maps as the remainder standard deviation after NS. Thereafter, one‐tailed paired *t*‐tests (*p* < 0.05) were performed on visual stimulation vs resting maps within anatomical ROIs defined by parcellation.

All processing steps were implemented as a batch using MATLAB 2021. Further details can be found in Supplementary material 2. The source codes for analysis are openly available at https://github.com/milecap/RFR_NS_pipelines.

## 3 RESULTS

### 3.1 RFR analysis

**FIGURE 2:**
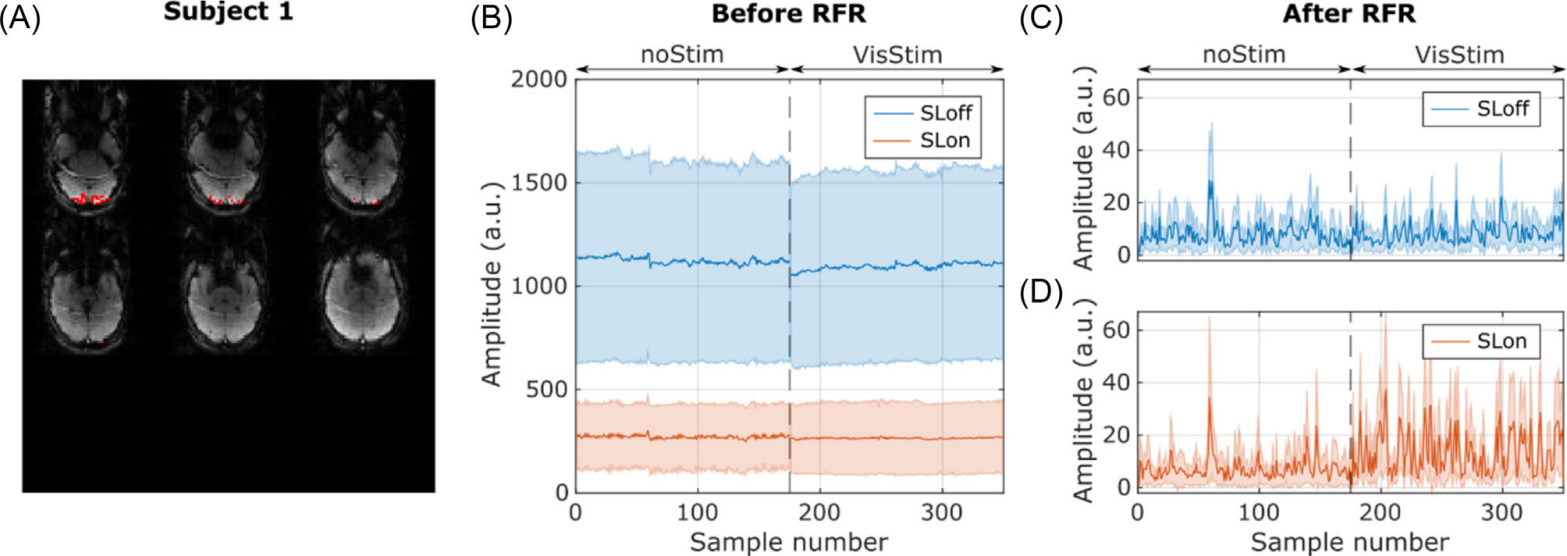
Impact of regression‐filtering‐rectification (RFR) procedure on signal time course of subject 1. (A) Overlay of occipital pole V1 on the SIRS acquisition; 55 voxels are included in the V1 ROI. (B) Time series of SLoff and SLon signals for nonstimulated and visually stimulated periods averaged in V1 ROI of the subject displayed in (A). (C and D) Output of the RFR procedure for resting and visual stimulation periods in SLoff and SLon signals, respectively. For (B), (C), and (D), the plain line represents the mean and the shaded area represents the standard deviation within the analyzed ROI. ROI, region of interest; SIRS, stimulus‐induced rotary saturation; SL, spin‐lock.

Figure 2A shows the overlay of the anatomically defined occipital pole on the SLoff acquisition of the SIRS sequence for one representative subject with significant visual activation (subject 1). Chemical shift artifacts are visible, but they remain constant over time and do not contribute to signal variance, and thus are not identified in activation maps. Figure 2B shows the four signals averaged within the voxels of the occipital pole as a function of time for the same subject. The standard deviation of the raw data comes from the contrast variance within the region of interest (ROI). Figure 2C and D compare the signals of the resting and stimulation periods after RFR procedure for the SLoff and SLon signals, respectively. The mean of the rectified signal is significantly higher (*p* < 0.05) for SL during visual stimulation compared to the rest period. There is no significant difference for the SLoff signal. Time courses in the V1 ROI can be observed for other significant and nonsignificant examples in Supplementary material 3.

**FIGURE 3:**
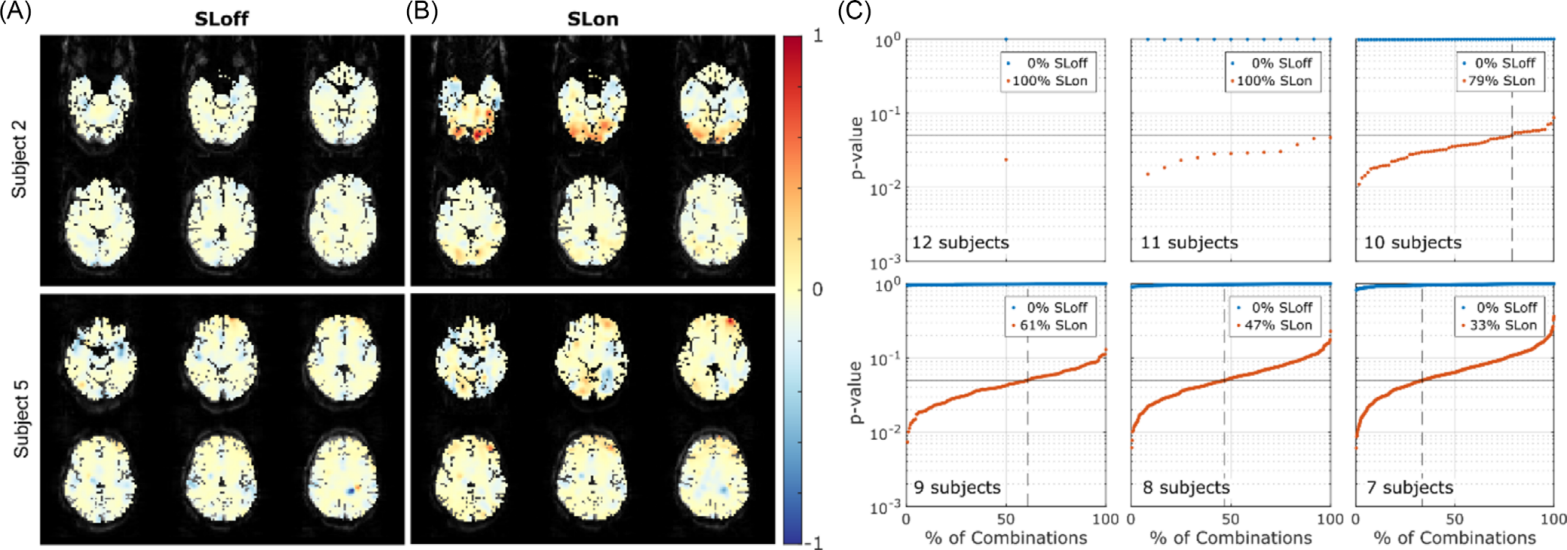
RFR results. Normalized contrast maps (VisStim–noStim) of two representative subjects after RFR for (A) SLoff and (B) SLon. Positive contrast difference is present in both subjects around the visual cortex, but only subject 2 presents significant activation (*p* < 0.05) in the occipital pole V1. (C) *p*‐values from one‐tailed *t*‐tests for visual stimulation vs. nonstimulation periods performed on 12, 11, 10, 9, 8, and 7 subjects. Values are sorted in ascending order for all combinations of subjects. The *t*‐test compares the mean of the rectified signal (proportional to signal variance after RFR) in both stimulation conditions. The label indicates the percentage of significant combinations found for both SLon and SLoff signals. RFR, regression‐filtering‐rectification; SL, spin‐lock.

Generally, no activation is observed in the contrast maps of the SLoff acquisitions (Figure 3A, both subjects). Instead, higher contrast areas are present in the SLon acquisitions, particularly in the occipital area (Figure 3B). The group analysis performed across all 12 subjects showed significant activation for the SLon acquisition (*p* = 0.02), and not for the SLoff (*p* = 0.99). Across all combinations of 11, 10, 9, 8, and 7 subjects, significant activation (*p* < 0.05) was found for 100%, 79%, 61%, 47%, and 33% of the combinations, respectively (Figure 3C). No significant activation was detected in the SLoff acquisition for any group size combination, indicating that the hemodynamic effect is not generating a significant contrast.

Individual statistical tests revealed that SLon acquisitions showed significant activation (*p* < 0.05) for the RFR contrast in four out of 12 subjects (33%). Instead, no significant contrast prevailed for SLoff acquisitions after RFR (example Figure 3, subject 2). Activation was visually observed in four additional subjects but did not reach statistical significance (*p* < 0.05) (example Figure 3, subject 5, other three subjects shown in Supplementary material 4). Negative contrast areas are observed in the contrast maps of a few subjects (example Figure 3); that is, the variance in these areas was greater at rest than during stimulation.

We analyzed the RFR output in specific anterior brain regions not involved in visual signal processing, including the subcallosal gyrus and the anterior segment of the circular sulcus of the insula, as previously suggested.^36^ No activation was found on SLon over the subcallosal gyrus, and only one subject showed significant false positive activation in the circular sulcus of the insula (*p* = 0.004). For this subject, a peak is observed in the SLon time course; therefore, activation was not associated with constant variance during stimulation, as demonstrated during a true SIRS effect. Activations outside the visual cortex appear in regions with low signal‐to‐noise ratio (SNR) or significant artefacts and can therefore be visually discarded.

**FIGURE 4:**
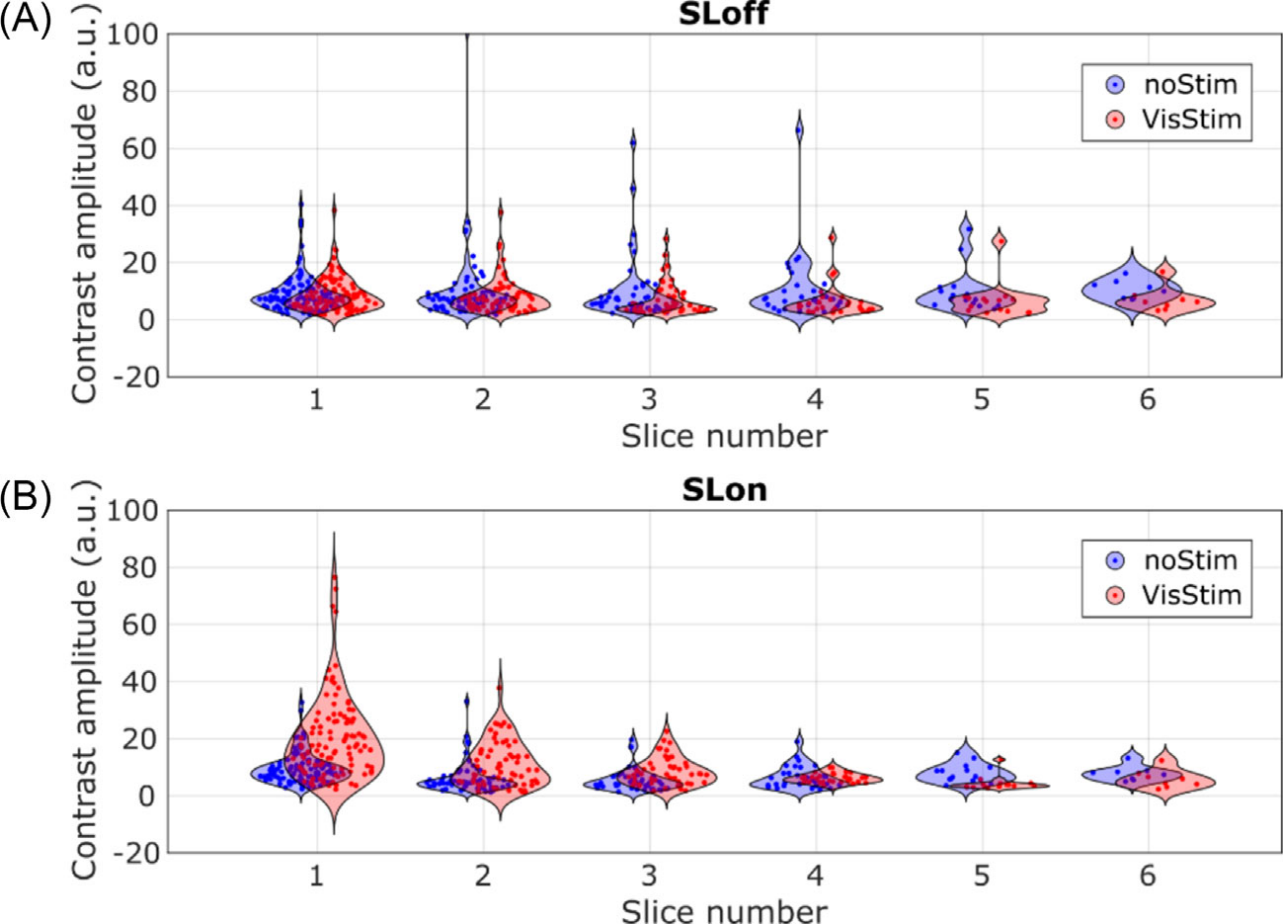
Global contrast dependence with slice number. (A) SLoff and (B) SLon contrast amplitude distributions for stimulated and nonstimulated conditions in the six acquired slices. Each point is given by the contrast in each voxel inside the occipital pole of all the subjects with significative contrast in the V1 ROI for each separate slice. The *x* positions are jittered for better visualization of the contrast distribution. ROI, region of interest; SL, spin‐lock.

Figure 4 shows the RFR output values within the voxels of the occipital pole for all subjects with significative contrast (subjects 1 to 4) as a function of the slice number. The *x* positions of the data points are jittered to better represent distribution along the *y*‐axis. The distributions show similar shapes for all slices in the SLoff acquisition (Figure 4A), while noticeable differences are observed especially in the first three slices of the SLon measurements (Figure 4B). The decay of signal variance difference between stimulated and rest conditions with the number of slices is attributed to $T_{1}$ relaxation. A different representation of this plot where the mean and std are easily observable is presented in Supplementary Material 5.

#### 3.1.1 ANOVA analysis of RFR output

**FIGURE 5:**
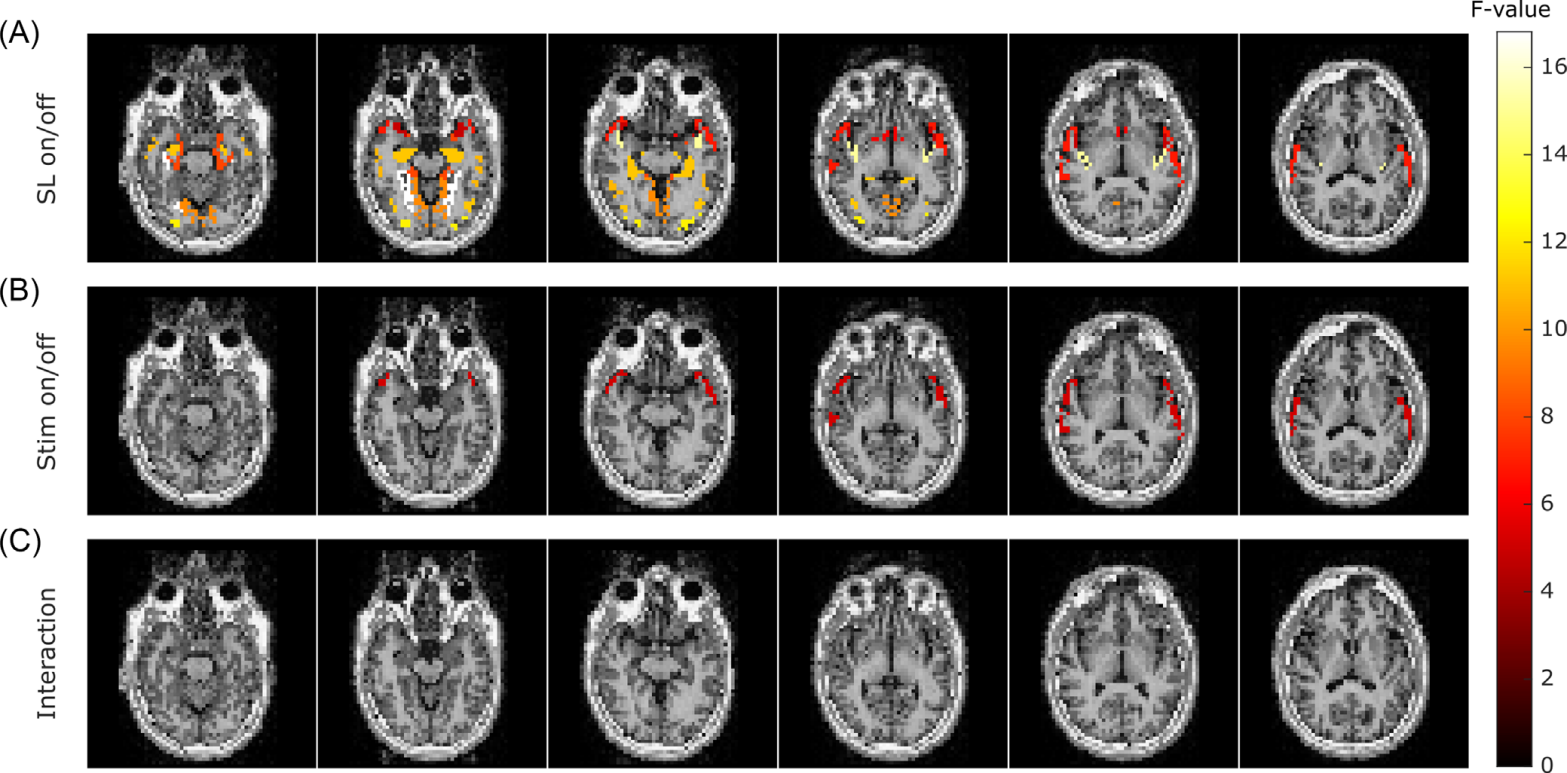
*F*‐value from the analysis of variance at the group level for each parcel of the Destrieux atlas with significative activation (*p* < 0.05) for three factors: (A) SL on/off, (B) Stim on/off, and (C) interaction term between SL and Stim conditions. SL, spin‐lock.

Due to the high variability of the individual results, we performed a two‐way ANOVA to assess the effect of the SL application, visual stimulation, and their interaction. Figure 5 displays the *F*‐values for the three factors in areas with significant activation (*p* < 0.05). The ANOVA revealed no significant effect of the visual stimulation or interaction terms, with only the SL factor showing group‐level activation. These activations are primarily localized in areas prone to low signal or artifacts.

### 3.2 NS analysis

**FIGURE 6:**
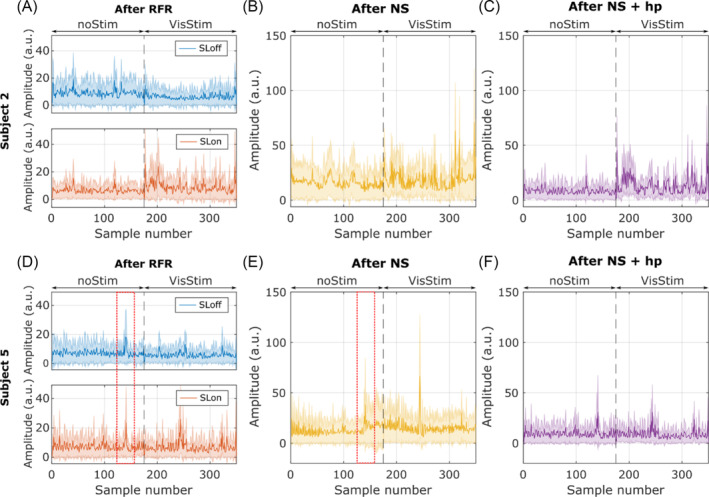
Comparison of NS and RFR procedures. (A and D) Output of the RFR procedure in the V1 occipital pole for subjects 2 and 5, respectively. (B and E) Output of the normalized subtraction for both subjects. The visible drift in the NS output of the VisStim condition leads to a false positive detection in subject 5. The dashed red lines highlight a spurious peak encoded in both SLon and SLoff acquisitions that is partially removed by NS. (C and F) output of the NS pipeline after the application of a high‐pass filter. NS, normalized subtraction; RFR, regression‐filtering‐rectification; SL, spin‐lock.

Statistical analysis on the occipital pole using the NS procedure detected significant activation (*p* < 0.05) in four out of 12 subjects (subjects 2, 4, 5, and 6). However, visible drift remained in the signal after NS in the four subjects, which was corrected by applying a high‐pass filter with a cutoff frequency of 0.1 Hz. This correction retained significant contrast in two subjects (2 and 4) who also showed activation after the RFR procedure. Figure 6A–C display both the RFR and NS output for subject 2, demonstrating significant activation in both pipelines. Figure 6D–F illustrate the false positive case observed in subject 5, where low‐frequency variations persisted after NS but not after high‐pass filtering. On the other hand, peaks present in both SLon and SLoff acquisitions significantly contributed to the variance of the RFR output (marked with a red square), with their relative importance being lower in NS. Supplementary material 6 displays the other two subjects of interest for the NS procedure.

## 4 DISCUSSION

This pilot study investigates whether periodic visual stimulation induces a measurable increase in signal variance following the application of a resonant SL preparation. Using the analysis pipeline outlined by Truong et al.,^33^ we observed a significantly higher variance during stimulation in the SLon condition compared to SLoff. However, the percentage of significant combinations decreases within smaller subgroups, consistent with findings by Truong et al. using a variation of SL targeting neuroelectric magnetic oscillations (NEMO), which we will refer to as the NEMO technique.^36^ NEMO, which operates on the same resonance principle but omits the final 90° tip‐up pulse, encodes the *z*‐projection of the magnetization after deviation from the SL‐direction. Because the expected deviation angle is small due to the small neuronal field amplitude, NEMO should yield higher sensitivity to the double resonance effect.^19^ Supplementary material 7 quantitatively illustrates the lower sensitivity depicted with the RESL‐SIRS technique compared to NEMO at the group level. Of note, the differences in the reported values may also reflect variations in the strength of the stimuli and differences in experimental design. The previous NEMO study targeted alpha activity on a block design, whereas our study involved continuous exposure to flickering light compared to resting baseline.

The decreasing significance rate in smaller subject groups indicates significant across‐subject variability. Individual analyses revealed significant activation in four subjects. The low detection rate can be attributed to several factors: first, the low sensitivity of the SIRS preparation, which has prompted the creation of new approaches to enhance sensitivity.^19^, ^36^, ^39^ Notably, positive detections using this technique have primarily been observed in epilepsy,^25^, ^27^ where neuronal activity exhibits significantly larger amplitudes than in healthy volunteers during visual stimulation. Second, sensitivity may be influenced by the primary evoked response occurring at the second harmonic of the stimulation frequency (16 Hz), although significant activation at the fundamental frequency has been reported in MEG and EEG studies.^40^, ^41^ Finally, SIRS is exclusively sensitive to the *z*‐component of magnetic fields. This study assumes broadly distributed fiber directions in the visual cortex, generating local magnetic fields with some *z*‐component, potentially impacting sensitivity, and causing false negative results.

Activation outside the visual cortex in Figures 3 and S4 may be attributed to the susceptibility of the RESL preparation to the nonuniform $B_{0}$ across the brain. This susceptibility can amplify signal variability,^9^ leading to false activations in high‐inhomogeneity regions. To avoid confounding effects in future studies, we suggest preparations that compensate for field inhomogeneities, such as CRESL^18^ or Balanced.^20^ Furthermore, combined approaches with other electrophysiological measures, such as EEG/MEG, need to be implemented to gain a better understanding of the signal response.

Whole brain coverage remains a challenge with this technique, with most previous methodological studies using a single‐slice acquisition.^20^, ^36^ Our study detected contrast across multiple slices despite $T_{1}$ attenuation (Figure 4), but a three‐slice restricted slab could yield similar results with a shorter TR. Two limitations of the employed approach are the reduced sample rate due to the long TR and the nonuniform sensitivity between slices caused by $T_{1}$ attenuation immediately diminishing contrast after preparation. Future experiments should explore fast 3D or multi‐slice acquisitions to address these limitations.

Following the analysis method proposed by Truong et al., we observed group‐level activation in the SLon condition but not in SLoff. However, a two‐way ANOVA revealed that the variability introduced by visual stimulation was negligible compared to the variance induced by the SL application. This suggests that the SLoff measurement may not serve as a robust control for SLon acquisitions. The higher variance of the SL measurement can therefore not be solely attributed to visual stimulation. The additional variance may result from $T_{1\rho }$ or BOLD fluctuations influenced by visual stimulation, which might be encoded by the SL preparation. To address this, future studies should incorporate control measurements using off‐resonance SL frequencies. Adapting this control approach to epilepsy remains challenging, as neural activity at other frequencies cannot be ruled out.

Regarding post‐processing pipelines, no significant contrast prevailed in the SLoff acquisition of any subject after RFR, indicating the proposed method's efficacy in removing physiological and hemodynamic confounds that contribute to signal variance. Generally, no low‐frequency changes were detected in the RFR output. Randomly emerging peaks were observed in SLon and SLoff acquisitions after RFR (Figures 2C and 6B), potentially leading to false positive or false negative results depending on their timing. The alternating acquisition of SLon and SLoff measurements allows for the detection of such artifacts at the cost of prolonging the effective repetition time. We have therefore initiated further experiments to determine the optimal acquisition method. Normalized subtraction reduced the significance of these peaks, while low‐frequency effects (e.g., drift, Figure 6C) persisted. The NS can remove spurious processes encoded in the common readout that vary more slowly than TR_eff_, suggesting that the variance metric can benefit from the combined application of both processing methods.

In conclusion, although there is evidence of increased variance in the stimulation condition for the SL preparation at the group level, the substantial variability introduced by the SL preparation itself, coupled with the absence of a robust control condition, prevents us from definitively attributing the detected activations to the SIRS contrast. This underscores the need for more sensitive and reliable SL preparations, as well as the establishment of better control measurements. As it stands, the current experimental approach and analysis pipeline do not reliably detect physiological responses to visual stimulation. The implementation of SL preparations that are robust to field inhomogeneity and faster readout methods are necessary for the further development and application of this technique.

## CONFLICT OF INTEREST STATEMENT

The authors declare no conflicts of interest.

## ETHICS STATEMENT

The study was approved by the Ethics Committee of the Medical University of Vienna.

## Supporting information


**Figure S1:** Detailed acquisition sequence expanded from Figure 1C in main text.
Figure S2: Pipeline implementation diagram. The full pipeline includes Acquisition, preprocessing, postprocessing and statistical comparison. The two used pipelines only differ in the postprocessing part and their output. The RFR procedure produces four maps while the NS produces two.
Figure S3: Occipital pole ROI overlaid over functional low‐resolution image (left) and time course after RFR procedure (right) for three subjects with significant (p < 0.05) activation in the occipital pole (A, B and C), and for three subjects with no significant activation in the occipital pole (D, E and F). The number of voxels within the V1 ROI for each subject where: A)121 B)35 C)64 D)84 E)111.
Figure S4: Normalized contrasts maps (VisStim–noStim) of subjects with significant (A, B and C) non‐significant (D, E and F) activation after RFR for SLoff and SLon. For Subjects 9, 10 and 11, positive contrast difference is observed in the in the visual cortex, but they did not reach statistical significance. In addition, areas of negative contrast, where the SLon variation was bigger during the rest period, can be observed.
Figure S5: Global contrast dependence with slice number. A) SLoff and B) SLon contrast amplitude distributions for stimulated and non‐stimulated conditions in the six acquired slices.
Figure S6: Comparison of RFR and NS output for subjects with positive detection in NS. A) and D) Output of the RFR procedure for subjects 4 and 6, respectively. B) and E) NS output for the same two subjects. Visible signal drift is observed in both subjects after NS. C) and F) output of NS procedure after high pass filter. The signal drift is corrected, eliminating the significant finding in subject 6.
Figure S7: Comparison of NEMO and SIRS results. Percentage of combinations in which significative difference between stimulation and resting state as a function of the population percentage. SIRS results correspond to the presented work, NEMO results correspond to the presented by Truong et al.

## Data Availability

Data available on request due to privacy/ethical restrictions. The source code used for the analysis presented on this study is openly available upon publication on GitHub, accessible at https://github.com/milecap/RFR_NS_pipelines.
