# Supplementary material for: Stimulus‐induced rotary saturation imaging of visually evoked response: A pilot study
Source: NMR Biomed. 2024 Nov 4;38(1):e5280. doi: 10.1002/nbm.5280 (PMC11602267; doi:10.1002/nbm.5280)
Supplement: Supplementary file 1 — Figure S1: Detailed acquisition sequence expanded from Figure 1C in main text. Figure S2: Pipeline implementation diagram. The full pipeline includes Acquisition, preprocessing, postprocessing and statistical comparison. The two used pipelines only differ in the postprocessing part and their output. The RFR procedure produces four maps while the NS produces two. Figure S3: Occipital pole ROI overlaid over functional low‐resolution image (left) and time course after RFR procedure (right) for three subjects with significant (p < 0.05) activation in the occipital pole (A, B and C), and for three subjects with no significant activation in the occipital pole (D, E and F). The number of voxels within the V1 ROI for each subject where: A)121 B)35 C)64 D)84 E)111. Figure S4: Normalized contrasts maps (VisStim–noStim) of subjects with significant (A, B and C) non‐significant (D, E and F) activation after RFR for SLoff and SLon. For Subjects 9, 10 and 11, positive contrast difference is observed in the in the visual cortex, but they did not reach statistical significance. In addition, areas of negative contrast, where the SLon variation was bigger during the rest period, can be observed. Figure S5: Global contrast dependence with slice number. A) SLoff and B) SLon contrast amplitude distributions for stimulated and non‐stimulated conditions in the six acquired slices. Figure S6: Comparison of RFR and NS output for subjects with positive detection in NS. A) and D) Output of the RFR procedure for subjects 4 and 6, respectively. B) and E) NS output for the same two subjects. Visible signal drift is observed in both subjects after NS. C) and F) output of NS procedure after high pass filter. The signal drift is corrected, eliminating the significant finding in subject 6. Figure S7: Comparison of NEMO and SIRS results. Percentage of combinations in which significative difference between stimulation and resting state as a function of the population percentage. SIRS results correspon [file NBM-38-e5280-s001.docx]

## Supplementary material 1: Acquisition details

A detailed expansion of Figure 1C of the main text is presented in Figure S1. The alternated SL off and SL on acquisitions all have the same duration. A TR is described as the addition of the preparation time (present even when no SL preparation is applied) followed by the readout time. The readout time consist of a 90° slice selective excitation (sinc pulse, duration 2.56 ms, time-bandwidth product 2.7) followed by an echo planar imaging readout phase of total duration 55 ms, and acquisition bandwidth 1950 Hz/pixel. No acceleration via parallel imaging was employed. No recovery delay is applied between SL off and SL on acquisitions, since for each slice total time of 5TR occurs before the following acquisition.


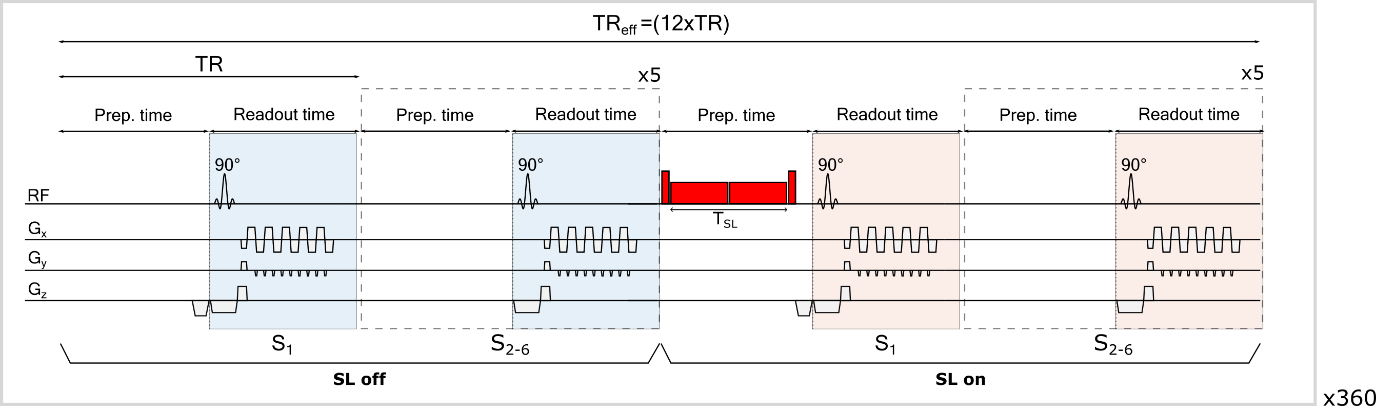


Figure S1: Detailed acquisition sequence expanded from Figure 1C in main text.

## Supplementary material 2: pipeline details

The magnitude EPI images directly extracted from the scanner without correction were used for this analysis. All the steps comprising the processing pipelines (normalized subtraction and regression-filtering-rectification) were implemented in MATLAB 2021 and are depicted in Figure S2. For both visual stimulation and resting series, we separated the alternated spin-lock (SL) on and off acquisitions and removed dummy scans. The first 10 scans were removed (first 5 SL off and first 5 SL on), as this allowed for the signal to stabilize after the stimulation onset. Motion correction was performed using FSL MCFLIR. We chose to utilize FSL for realignment due to its padding option, particularly useful when working with a limited number of acquired slices^1^. High resolution images were segmented using DL+DiReCT^2^, and SPM 12 was used to coregister the segmented anatomical images to the functional space. Subsequent analyses were performed within the functional space for both pipelines.


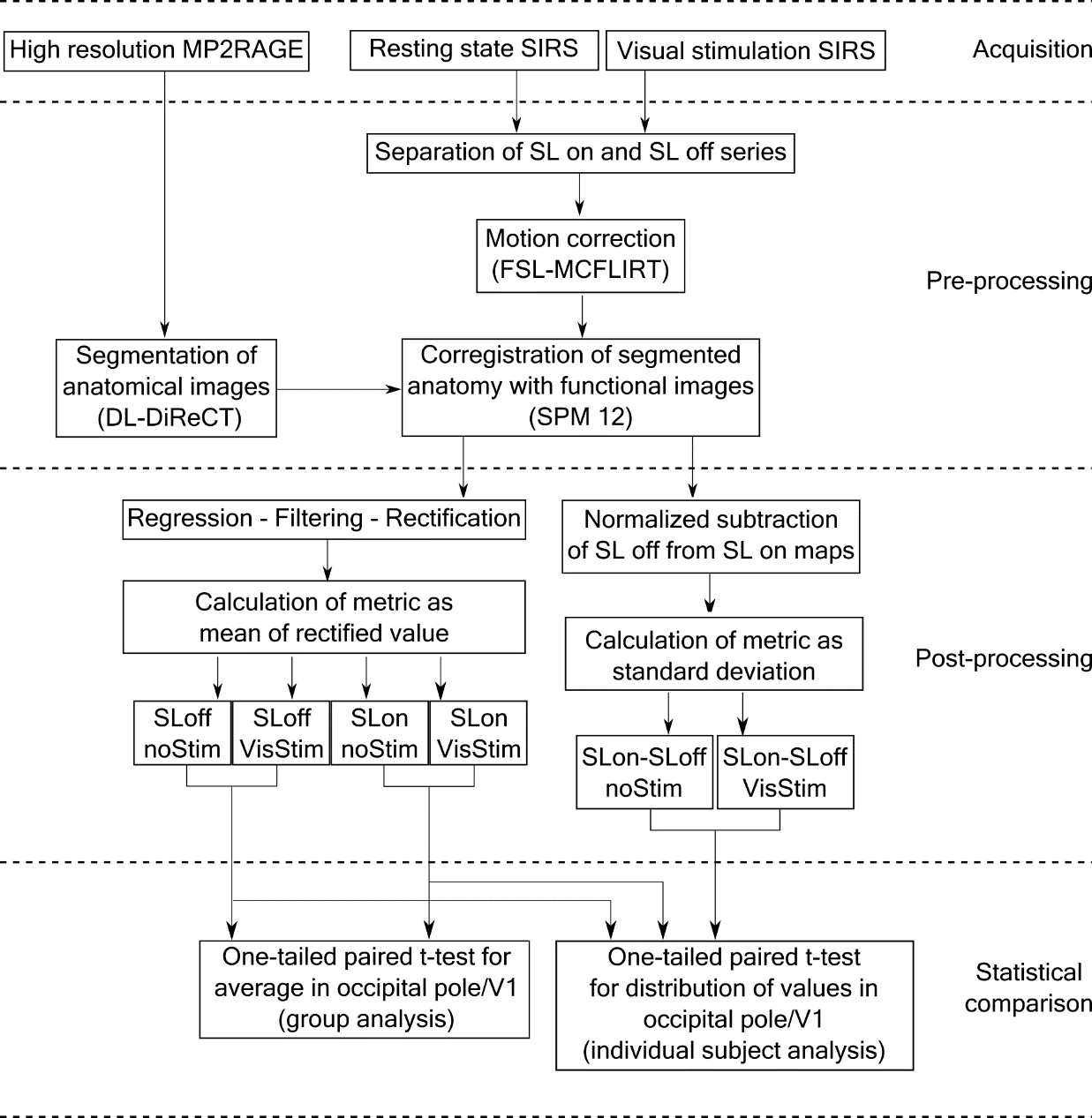


Figure S2: Pipeline implementation diagram. The full pipeline includes Acquisition, preprocessing, postprocessing and statistical comparison. The two used pipelines only differ in the postprocessing part and their output. The RFR procedure produces four maps while the NS produces two.

## Supplementary material 3: time-courses RFR

Impact of regression-filtering-rectification (RFR) procedure on signal time course for three subjects 2, 3 and 4 that displayed significant activation on the occipital pole are showed in Figure S3 A), B) and C) respectively. Examples of subjects that didn’t have significant activation (subject numbers 5, 6 and 7) are displayed in Figure S3 D), E) and F) respectively. It is possible to observe the low quality of the functional acquisition along with the difference in the total number of voxels included in the V1 ROI.


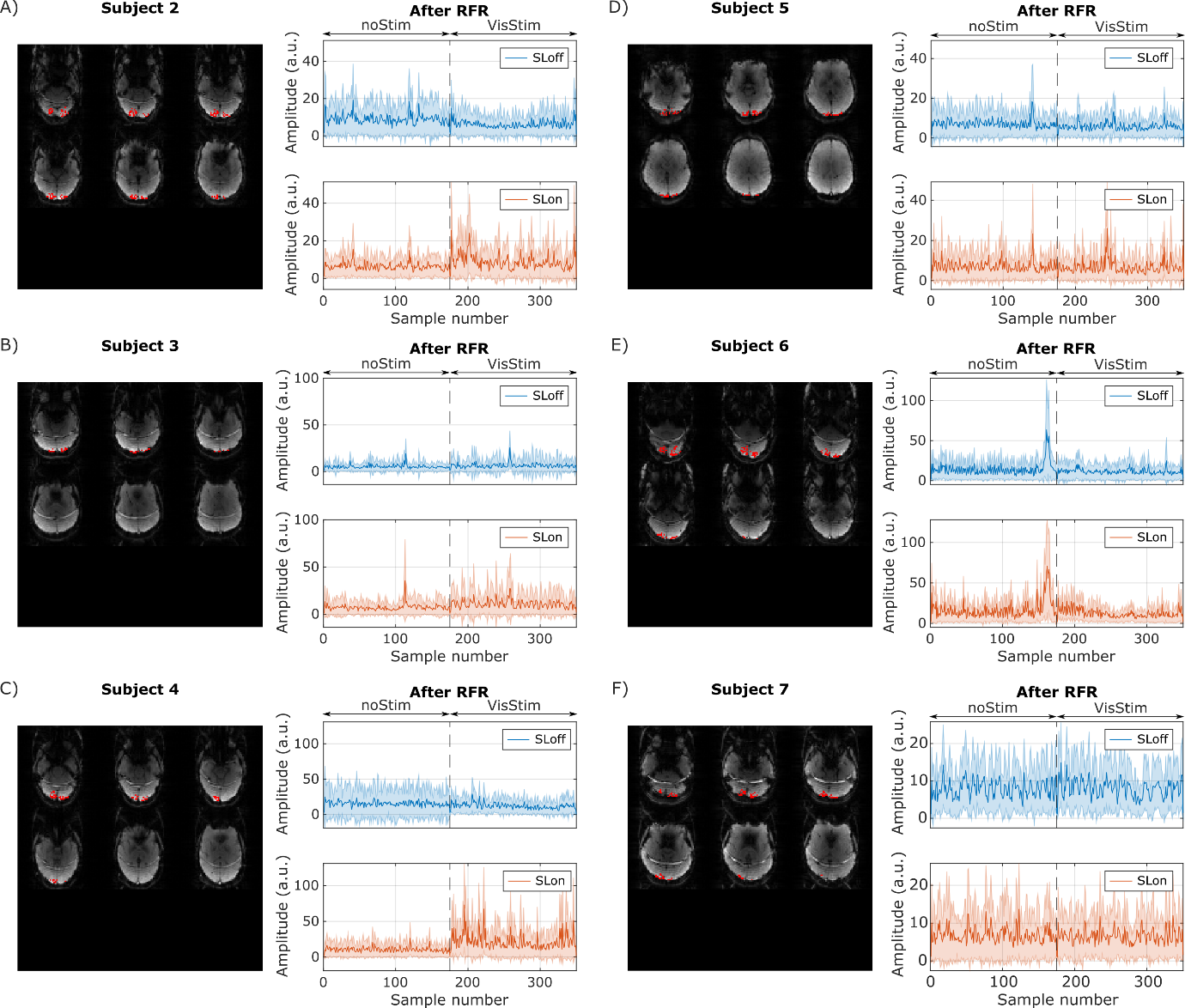


Figure S3: Occipital pole ROI overlaid over functional low-resolution image (left) and time course after RFR procedure (right) for three subjects with significant (p < 0.05) activation in the occipital pole (A, B and C), and for three subjects with no significant activation in the occipital pole (D, E and F). The number of voxels within the V1 ROI for each subject where: A)121 B)35 C)64 D)84 E)111.

## Supplementary material 4: contrast maps

The RFR processing pipeline identified significant activation in the occipital pole for 4 out of 12 subjects. One is shown in Figure 3 of the main text, the other three are shown in Figure S4 A, B and C. In addition, variations in the SLon signal are observed in other 4 subjects but did not reach statistical significance. One of these subjects is depicted in Figure 3 of the main text, while the other three cases with non-significant activations are shown in Figure S4 D, E and F. In all three cases, higher contrast is observed in the SL prepared maps, and the maxima are located near the visual cortex. However, spurious negative effects were also observed in these three subjects. It is important to note that when determining the significance of a specific ROI, averaging all the voxels within it can lead to false negatives due to these spurious negative signals. Hence, regardless of the post-processing technique employed, visual observation of the signal is still necessary.


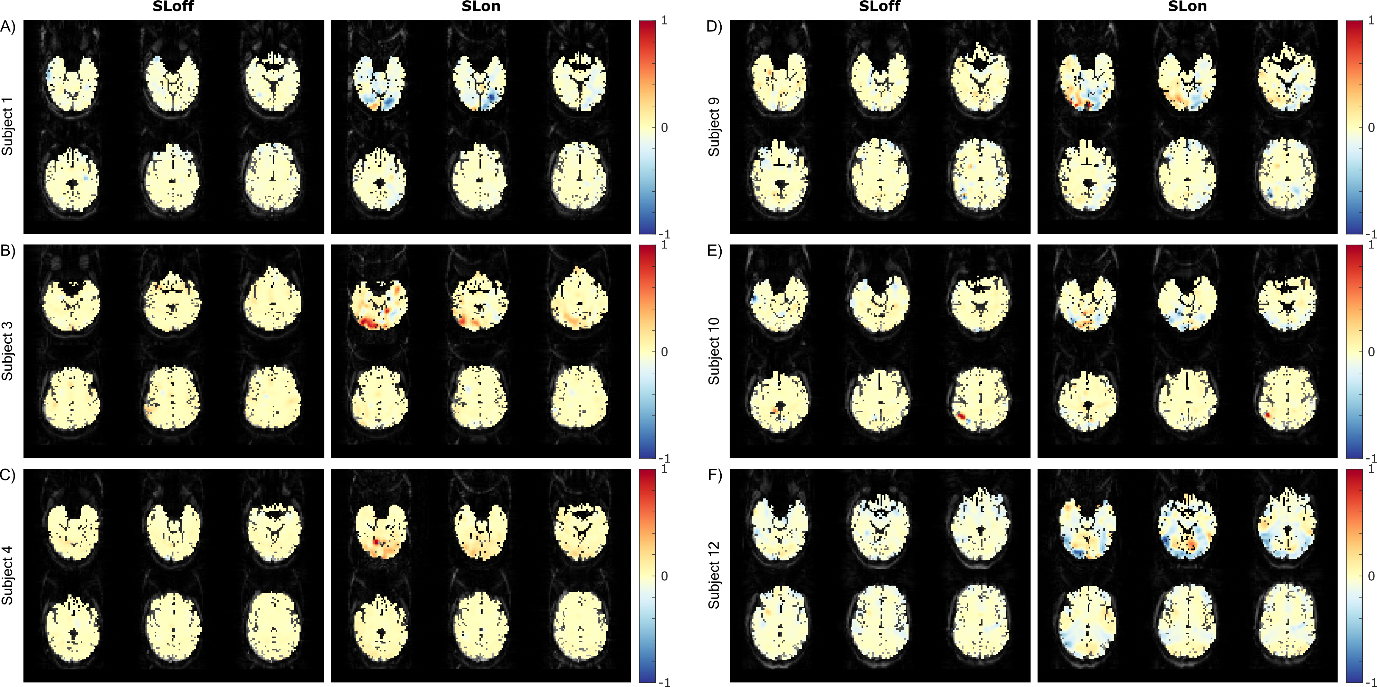


Figure S4: Normalized contrasts maps (VisStim – noStim) of subjects with significant (A, B and C) non-significant (D, E and F) activation after RFR for SLoff and SLon. For Subjects 9, 10 and 11, positive contrast difference is observed in the in the visual cortex, but they did not reach statistical significance. In addition, areas of negative contrast, where the SLon variation was bigger during the rest period, can be observed.

## Supplementary material 5: Global contrast dependance

Figure 4 shows the RFR output values within the voxels of the occipital pole for all subjects with significative contrast (subjects 1 to 4) as a function of the slice number.


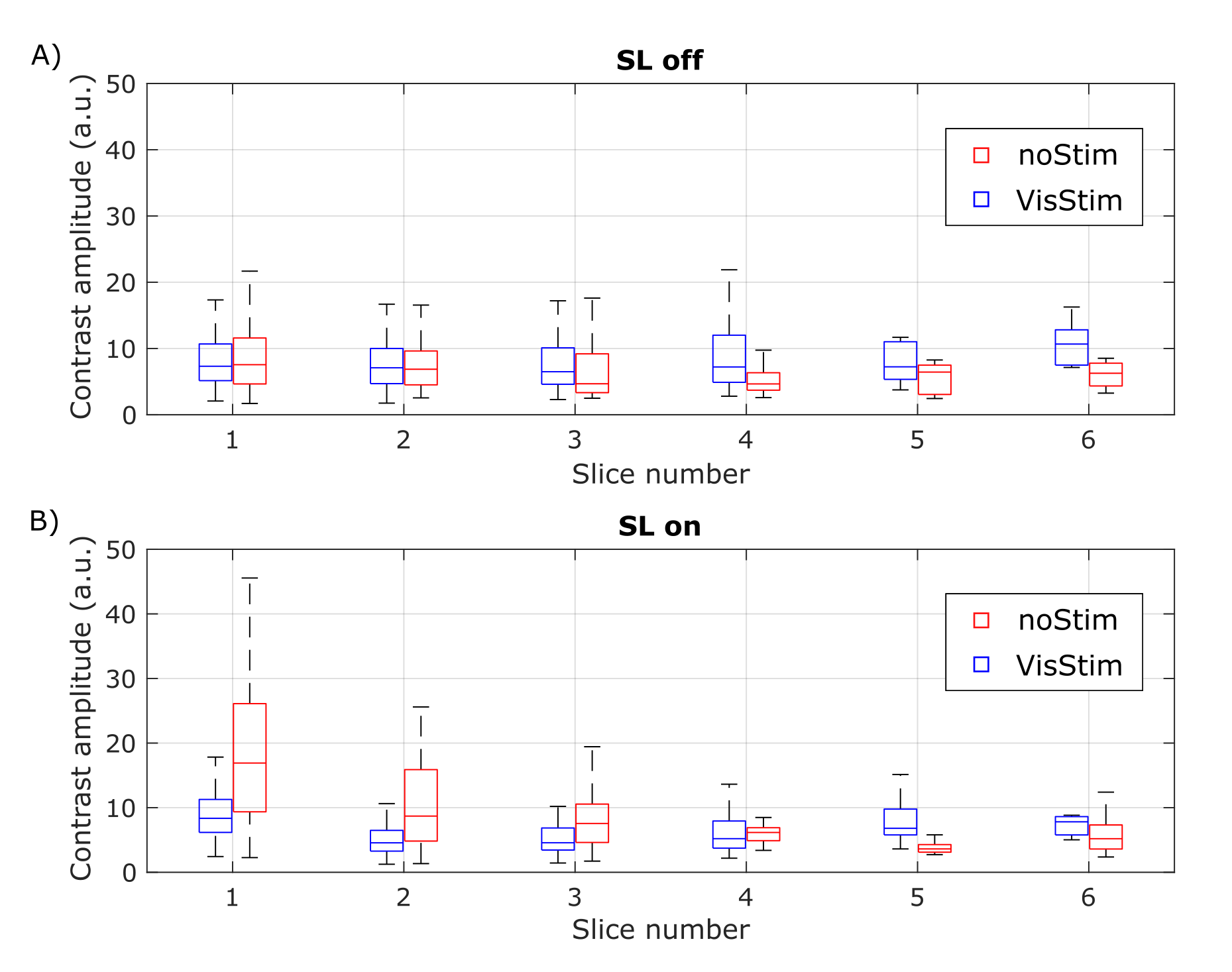


Figure S5: Global contrast dependence with slice number. A) SLoff and B) SLon contrast amplitude distributions for stimulated and non-stimulated conditions in the six acquired slices.

## Supplementary material 6: NS output

Figure S6 shows two cases that displayed significant activation in the visual cortex after the NS procedure (Subjects 4 and 6), identically as it was displayed in Figure 5 of the main text for subjects 2 and 5. The RFR output, the NS output and the Ns output after high pass filter are displayed. Subject 4 presented significant contrast for both RFR and NS + high pass pipelines. Instead, subject 6 was only significant for the plain NS pipeline, but not for RFR nor NS + high pass, showing the need to apply the filter to eliminate low frequency variations in the NS procedure.


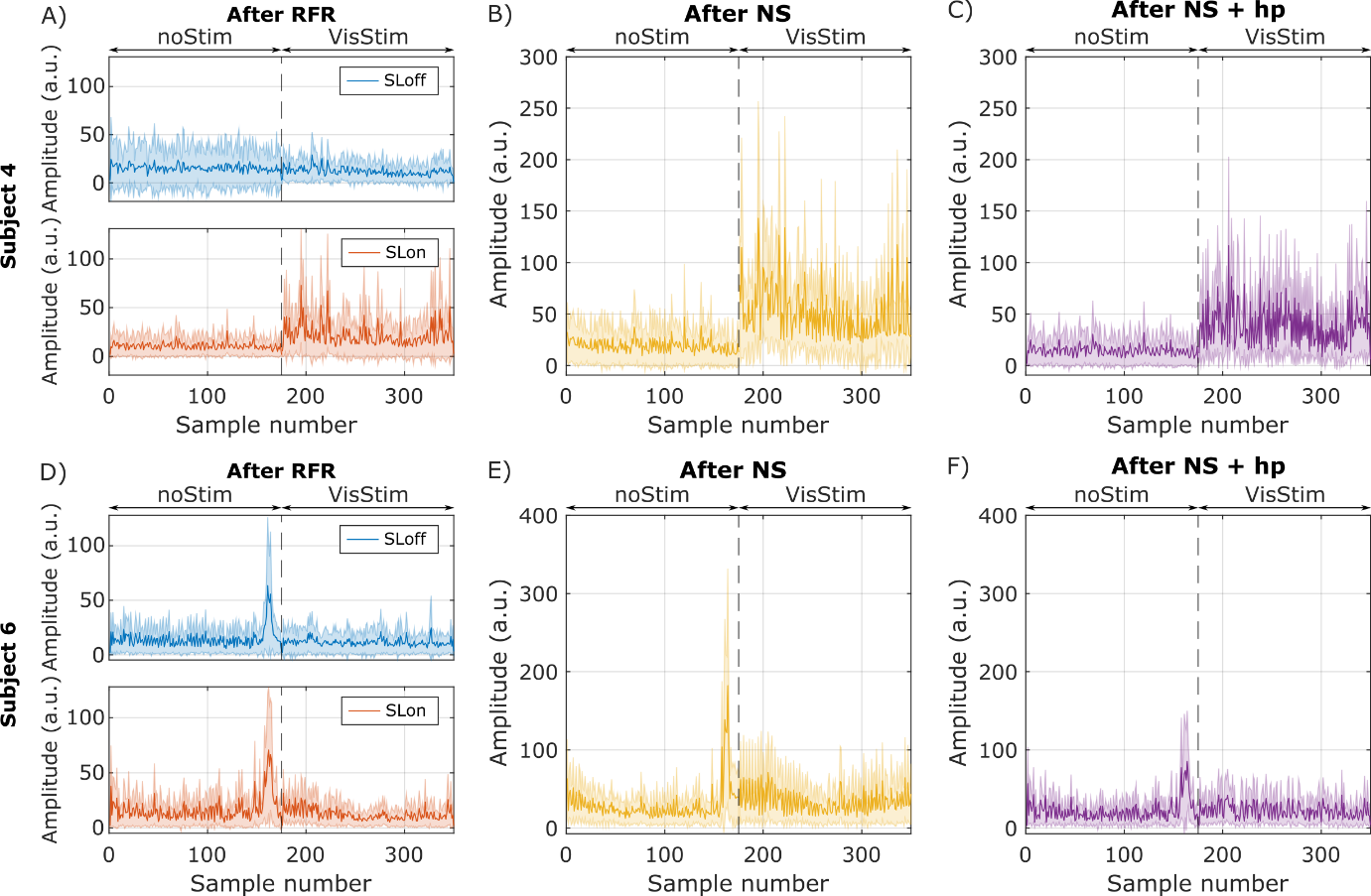


Figure S6: Comparison of RFR and NS output for subjects with positive detection in NS. A) and D) Output of the RFR procedure for subjects 4 and 6, respectively. B) and E) NS output for the same two subjects. Visible signal drift is observed in both subjects after NS. C) and F) output of NS procedure after high pass filter. The signal drift is corrected, eliminating the significant finding in subject 6.

## Supplementary material 7: Group analysis and pipeline comparison

To the best of our knowledge, the only previously published work on the in-vivo rotary saturation effect did not report the absolute percentage of individuals in whom activation was detected (33% for this study). Instead, results are presented for a group analysis^3^. To compare the performance of our method with the NEMO sequence used in that study, we performed the same group analysis. For this, the mean metric was averaged over all voxels in the anatomically defined occipital pole/V1 ROI of each subject, and t-tests were performed for all possible combinations over 11, 10, 9, 8 and 7 of the 12 subjects as described in the main text. A one-tailed t-test was performed to assess whether the visual stimulation series had a higher mean than the no-stimulation period. We then can compare the percentage of combinations that was detected for each combination. Results are shown in Figure S7. When taking all the subjects in both methods, they both result in a significant contrast (p<0.05), showing that both methods can detect visual activation. However, for smaller groups, the percentage of detected activations is lower for SIRS than for NEMO, which is expectable giving the lower sensitivity of the used SL technique.


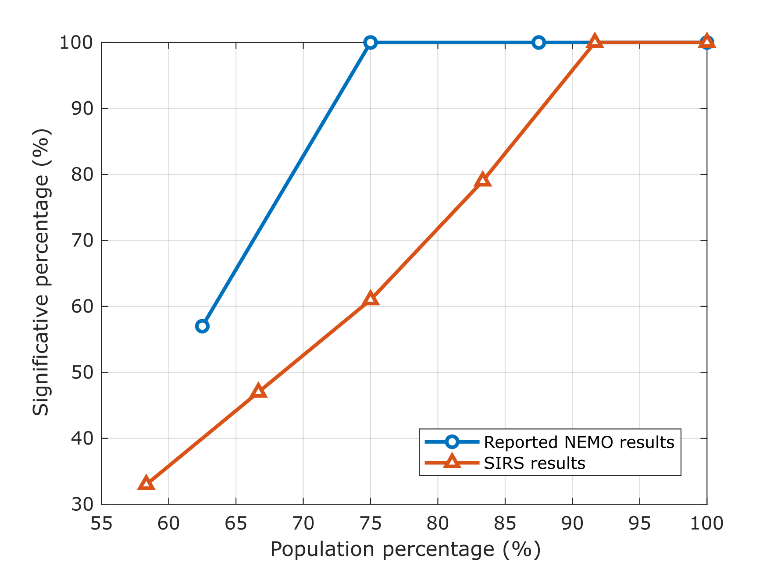


Figure S7: Comparison of NEMO and SIRS results. Percentage of combinations in which significative difference between stimulation and resting state as a function of the population percentage. SIRS results correspond to the presented work, NEMO results correspond to the presented by Truong et al.

1. Jenkinson M, Bannister P, Brady M, Smith S. Improved Optimization for the Robust and Accurate Linear Registration and Motion Correction of Brain Images. *Neuroimage*. 2002;17(2):825-841. doi:10.1006/NIMG.2002.1132

2. Rebsamen M, Rummel C, Reyes M, Wiest R, McKinley R. Direct cortical thickness estimation using deep learning-based anatomy segmentation and cortex parcellation. *Hum Brain Mapp*. 2020;41(17):4804-4814. doi:10.1002/HBM.25159

3. Truong TK, Roberts KC, Woldorff MG, Song AW. Toward direct MRI of neuro-electro-magnetic oscillations in the human brain. *Magn Reson Med*. 2019;81(6):3462-3475. doi:10.1002/mrm.27654
